# Rapping Mania

**Published:** 1853-04

**Authors:** 


From the New York Medical Gazette.

*Rapping Mania.*

Our readers will bear us witness that from the modern inception of this terrible iniquity at Rochester, and the attempted mercenary speculation of transferring the "weird sisters" to this city to exhibit themselves to visitors at a dollar a head, this paper fully and fearlessly denounced all parties who gave the foul imposture its first impulse in New York, whether Clerical, Judicial, Legal or Medical, as the dupes of designing men and women conspired to deceive. We then took the early responsibility, not merely to deny anything supernatural or spiritual in the "mysterious knockings," but we boldly alleged a systematic scheme of villainy upon the women who were used, and the men who used them, to make money by deception, fraud, and imposture; and insisted that for obtaining money under false pretenses, all parties were open to conviction under our statute law, and we called on the District Attorney, Grand Jury and Courts, to do their duty; naming the Penitentiary as the due of the knaves, and predicting the Lunatic Asylum as the refuge of their dupes.

But finding our efforts fruitless, and our warning unheeded, and discovering that a portion of the public press had been subsidized to silence, and still another portion hired to echo the mystery, and give currency to the pretended revelations from the spirit-world, we renewed our exertions to arrest the unutterable mischiefs of the delusion. Foreseeing the certainty that multitudes would be led away by these deceivers, if they were not unmasked, and their knavery exposed, we sent forth a warning, not merely against the insanity which must result upon weak minds, but urging that the dethronement of reason which would follow would be suicidal and homicidal, and hence predicted deeds of "darkness, infamy and blood," as the legitimate and unavoidable results, of a belief in these spiritual and ghostly revelations.

It was but a few weeks before the knavery of the weird sisters, Fish and Foxes, was proved and promulgated by Professors Flint and Lee, of Buffalo, two members of our profession, who nobly threw themselves into the breach in the vain hope of stopping the plague; and their exposure of these prime movers of the rapping mania, has since been verified by the Cincinnati Ladies' Committee so as to annihilate every doubt. Meanwhile insanity has gone on multiplying, and filling up our Asylums all over the country with its victims, and thousands more are tottering on its verge, bewildered and confounded by morbid imaginings of ghostly messages from the spirit-world. The deeds of darkness, infamy and blood, justly apprehended as the fruits of this form of insanity, have repeatedly been published in the suicides and bloody murders of both men and women, leaving widows and orphans to bewail their hapless fate. And all this because summary punishment has not been inflicted upon the guilty originators and propagators of this abominable imposture.

So far from visiting these offenders with the penalties of the law, there are clergymen, physicians, judges, lawyers, and even editors, who have marvelled and doubted, and gravely said, there is something strange, mysterious, unaccountable in some of these revelations, and speculated on their causes; thus admitting the reality of the so-called phenomena, and conceding something supernatural, if not ghostly in them; and thus betraying all around them who do their thinking by proxy, into the belief of all the miracle-mongering, which the rappers and their victims allege.

Hence, they have gone on from one imposture to another, from rapping and alphabets when these become stale, to bell-ringing, table-moving, singing, dancing, writing, discerning spirits, healing diseases, revealing truths and denouncing errors in religion, morals, science and philosophy, and all professedly from the ghosts of the departed. And the public press has done, and is doing, much to perpetuate the iniquity, by recording as facts the most absurd of these stories. In several of these presses, the movement of tables has been alleged as capable of being effected by a circle sitting around it, and touching it with their hands; thus giving color to the wildest of the ghostly stories, while disclaiming these and alleging electricity as the cause. But they forgot to tell, that the tables only move in the *dark*, and that there will be found under each moving table a stout negro, white or black, whose muscles furnish the locomotion. If any body alledges the contrary, we have a small table in our office on which we write, and we offer one hundred dollars to any ghost or medium, from this world or the other, who will move it an inch in daylight, by any supernatural, spiritual, magnetic or electrical influence, which shall be invisible and intangible to our own optics; and they may sit in a circle around it for a month, and "call spirits from the vasty deep, but will they come?"

Surely, sense has "fled to brutish beasts, and men have lost their reason," who can be so easily gulled to sacrifice their intelligence, their phi'osophy, their religion, and even their Bibles, to the ghostly rappings of these impostors, and even "pay for the poker." But we have done our duty, and will only again express our gratification at the fact that no regular physician within our knowledge has been seduced from his propriety by this stupendous folly, and that Homœopathists are the only medical victims of the mania to which the honest among them have an intellectual proclivity, as they have ever had to the kindred impostures of animal magnetism. Thus far they have only been found among the knaves, and make money by the operation. We believe no one of them has yet become a dupe.

